# 

**Published:** 1865-12

**Authors:** 


### Books and Pamphlets Received.

On Wakefulness. With an Introductory Chapter on the Physiology of Selep. By William A. Hammond, M. D. Philadelphia: J. B. Lippincott & Co. 1866.

Chloroform: Its Action and Administration. By Arthur Ernest Sansom, M. B., London. Philadelphia; Lindsay & Blakiston. 1866.

Lectures on Epilepsy, Pain, Paralysis, and certain other Disorders of the Nervous System. By Charles Bland Radcliffe, M. D. Philadelphia: Lindsay & Blakiston. 1866.

On the Diseases, Injuries and Malformations of the Rectum and Anus; with Remarks on Habitual Constipation. By T. J. Ashton. With Illustrations. Second American from the fourth and revised English edition. Philadelphia: Henry C. Lea. 1865.

The Principles of Surgery. By James Syme, F. R. S. E. To which are appended his Treasises on "The Diseascs of the Rectum," "Stricture of the Urethra and Fistula in Perineo," "The Excision of Diseased Joints," and numerous additinal Contributions to the Pathology and Practice of Surgery. Edited by his former pupil, Donald Maclean, M. D., L. R. C. S. E. Philadelphia: J. B. Lippincott & Co. 1866.

The Practice of Medicine. By Thomas Hawkes Tanner, M. D., F. L. S. From the fifth London edition. Enlarged and improved. Philadelphia: Lindsay & Blackiston. 1866.

On Epidemic Cholera, the Phenomena, Causes, Prevention and Treatment. With an Appendix, relating to the Brooklyn City Sewerage. By Nelson L. North, M. D. Reprint from the Transactions of the Medical Association, of the Eastern District of Brooklyn.

Report by the Council of Hygiene and Public Health, of the Citizens' Association of New York, upon Epidemic Cholera and Preventive Measures. New York, November, 1865.

Introductory Lecture, delivered at the Opening of the New York College of Veterinary Surgeons, November 6, 1865. By Prof. A. S. Copeman.

Tenth Annual Report of the Trustees of the State Lunatic Hospital at Northampton. October, 1865.

Oration, delivered July 4th, 1865, at Eden, Erie County, N. Y. By James Sheldon.

